# Management and Follow-Up of a Traumatic Vertebral Artery Pseudoaneurysm: A Case Report on Pipeline Embolization and Recovery

**DOI:** 10.7759/cureus.84578

**Published:** 2025-05-21

**Authors:** Quintin Norris, Nicolas A Siegelman, Steven Leckie

**Affiliations:** 1 Orthopaedic Surgery, Nova Southeastern University Dr. Kiran C. Patel College of Osteopathic Medicine, Clearwater, USA; 2 Osteopathic Medical School, Nova Southeastern University Dr. Kiran C. Patel College of Osteopathic Medicine, Clearwater, USA; 3 Orthopaedic Surgery, Beth Israel Deaconess Medical Center, Plymouth, Plymouth, USA

**Keywords:** endovascular treatment, pipeline flow diverter embolization, post-traumatic bodily injury, radiculopathy mimic, rehabilitation and recovery, vertebral artery pseudoaneurysm

## Abstract

Pseudoaneurysms can present as a rare but important mimicry of radiculopathy, particularly in cases of progressive symptoms where there is no obvious spinal pathology. This report details the case of a 69-year-old male who developed a post-traumatic left vertebral artery pseudoaneurysm following a low-energy fall, which led to intensifying neck pain and significant left arm weakness. The large vertebral artery pseudoaneurysm was successfully managed using pipeline embolization, and the patient’s subsequent recovery is outlined, including the operative approach, post-operative progress, and follow-up care. The follow-up focused on the patient’s rehabilitation efforts, ongoing imaging results, and continuous monitoring for any complications. This case underscores the complexity of diagnosing traumatic vertebral artery pseudoaneurysms, particularly given the absence of comprehensive guidelines for their management and the often misleading presentation. The innovative use of pipeline embolization in this case, along with the patient's ongoing rehabilitation, highlights the potential of endovascular techniques in the treatment of such conditions and contributes valuable insight to the limited literature on vertebral artery pseudoaneurysms.

## Introduction

Pseudoaneurysms are areas of blood collection within a vessel that occur due to vascular injury leading to blood leakage in the surrounding tissues; they differ from true aneurysms, which involve dilation of all three layers of the vessel wall [1]. In a pseudoaneurysm, blood leaks from the injury site but remains enclosed by a fibrotic wall formed from clotting cascade products such as collagen and fibrin, rather than the vessel wall itself [1]. The formation of a pseudoaneurysm is commonly associated with high-energy trauma, such as falls or motor vehicle accidents, which cause damage to the arterial wall through direct injury, shear forces, or hyperextension [2-3]. In the cervical spine, the risk is exacerbated due to the proximity of the vertebral arteries to bony structures and the potential for compressive forces [4]. Additional factors, including prior surgical procedures or pre-existing spinal conditions, may also contribute to an increased risk of pseudoaneurysm formation [5]. These injuries are concerning due to their potential to cause severe neurological complications if not promptly identified and treated, making early recognition essential, as their subtle signs are often overlooked. Pseudoaneurysms of the vertebral artery are a form of posterior circulation stroke and have an annual incidence of 18/100,000 [6]. Previous management typically involved microscopic surgery, but recent advances, including covered endovascular stents, have increased the safety and efficacy of management. 

Pseudoaneurysms often present with non-specific or misleading symptoms, which lead to diagnostic challenges and delayed treatment [7]. In cases involving neck trauma, the symptoms can mimic more common conditions such as cervical radiculopathy or musculoskeletal injuries [8]. Without timely diagnosis, these vascular injuries can progress, increasing the risk of rupture or stroke [9]. Therefore, maintaining a high suspicion for pseudoaneurysms in patients with unexplained neurological deficits following trauma is essential for appropriate management and improved outcomes.

This case study delves into a rare and complex presentation of a vertebral artery pseudoaneurysm resulting from a seemingly minor neck injury. While cervical pseudoaneurysms are already a rare occurrence from such injuries, the complexity of the treatment plan was exacerbated by the subtle and misleading symptoms that initially mimicked cervical radiculopathy. This report highlights the intricacies of diagnosing and managing a pseudoaneurysm, emphasizing the importance of early detection and a multidisciplinary approach to treatment. The case demonstrates an effective strategy for addressing vascular injury while mitigating the risk of severe complications.

The patient was informed about the potential publication of the case details, including radiographic images, and provided their consent.

## Case presentation

A 69-year-old male patient presented with neck pain, left deltoid pain, and left hand weakness secondary to slipping and falling into a wall. On initial examination, there were no motor or sensory deficits aside from slight 4+/5 weakness in the left hand. Cervical X-rays showed no instability, acute fractures, or osteolytic lesions. The patient had no significant past medical history, including cardiac, pulmonary disease, cancer, or diabetes. Initial findings suggested cervicalgia, leading to a treatment plan of outpatient physical therapy, methocarbamol, and follow-up as needed.

**Figure 1:**
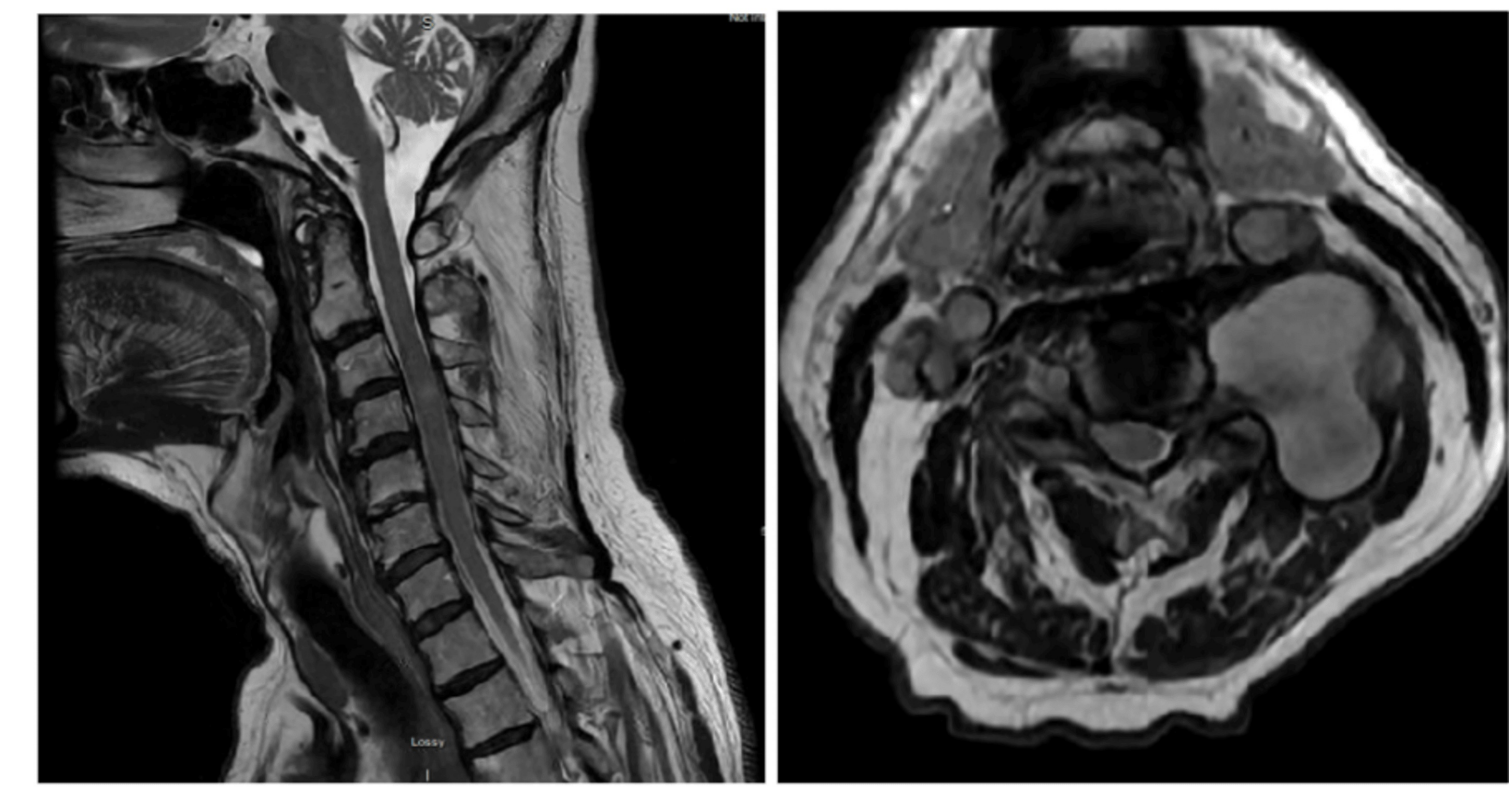
Magnetic Resonance Imaging (MRI) of cervical spine MRI of cervical spine reveals a lobular T2 hyperintense lesion adjacent to C4, measuring 4.1 x 3.1 x 4.1 cm, with heterogeneous T1 signal. The lesion extends towards the left vertebral artery. Notable findings include increased signal at C4, suggesting cord edema, and degenerative changes with varying degrees of central canal and foraminal stenosis at multiple cervical levels.

Three months later, he returned with progressive weakness of the left upper extremity and accompanying paresthesias. Upon further examination, the patient exhibited 0/5 strength in the left deltoid and biceps, with no other focal neurological abnormalities. An urgent cervical MRI was ordered and revealed a suspicious 4.1 x 3.1 x 4.1cm lesion to the left of the C4 vertebrae (Figure 1).

**Figure 2:**
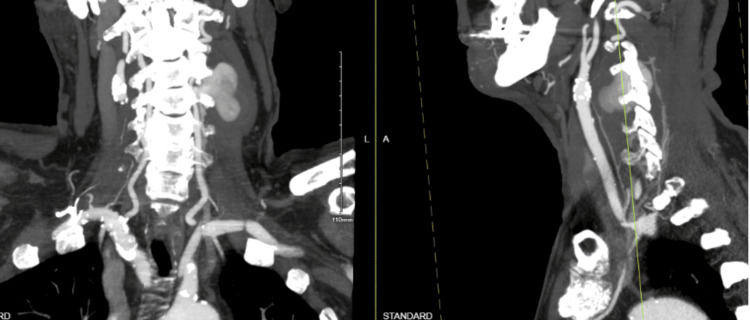
Computed tomography angiography (CTA) of neck CTA of neck demonstrated a paraspinal enhancing mass measuring 2.2 x 3.6 x 4.0 cm (tr x ap x cc) causing 75% narrowing of the left vertebral artery at the level of C2-C3 (2:176) likely representing a pseudoaneurysm with extension into the left intraspinal epidural space and the left paraspinal and displacing the paraspinal muscles.

Subsequent Computed Tomography Angiography (CTA) imaging of the neck confirmed a large 4.5 x 2.7cm left vertebral artery pseudoaneurysm of the V2 segment adjacent to C3-C4, extending into the left intraspinal epidural and paraspinal space. Additionally, there was evidence of right vertebral artery dissection extending from C2-C3 (Figure 2). Vascular surgery, neurosurgery, and neurology were all consulted and agreed with the plan of performing a diagnostic cerebral angiogram and considering flow diversion treatment for the left vertebral artery pseudoaneurysm.

The patient was positioned in the neurointerventional suite, and general anesthesia was administered. Using fluoroscopy and ultrasound, the right common femoral artery was accessed, and an 8 French sheath was placed with continuous heparinized saline flush. A 5 French Berenstein 2 catheter was advanced for a diagnostic cerebral angiogram, followed by roadmap guidance to advance a Navien intermediate catheter with a Phenom microcatheter and Aristotle microwire into the left proximal V2 segment, past the dissection flap and giant pseudoaneurysm.

**Figure 3:**
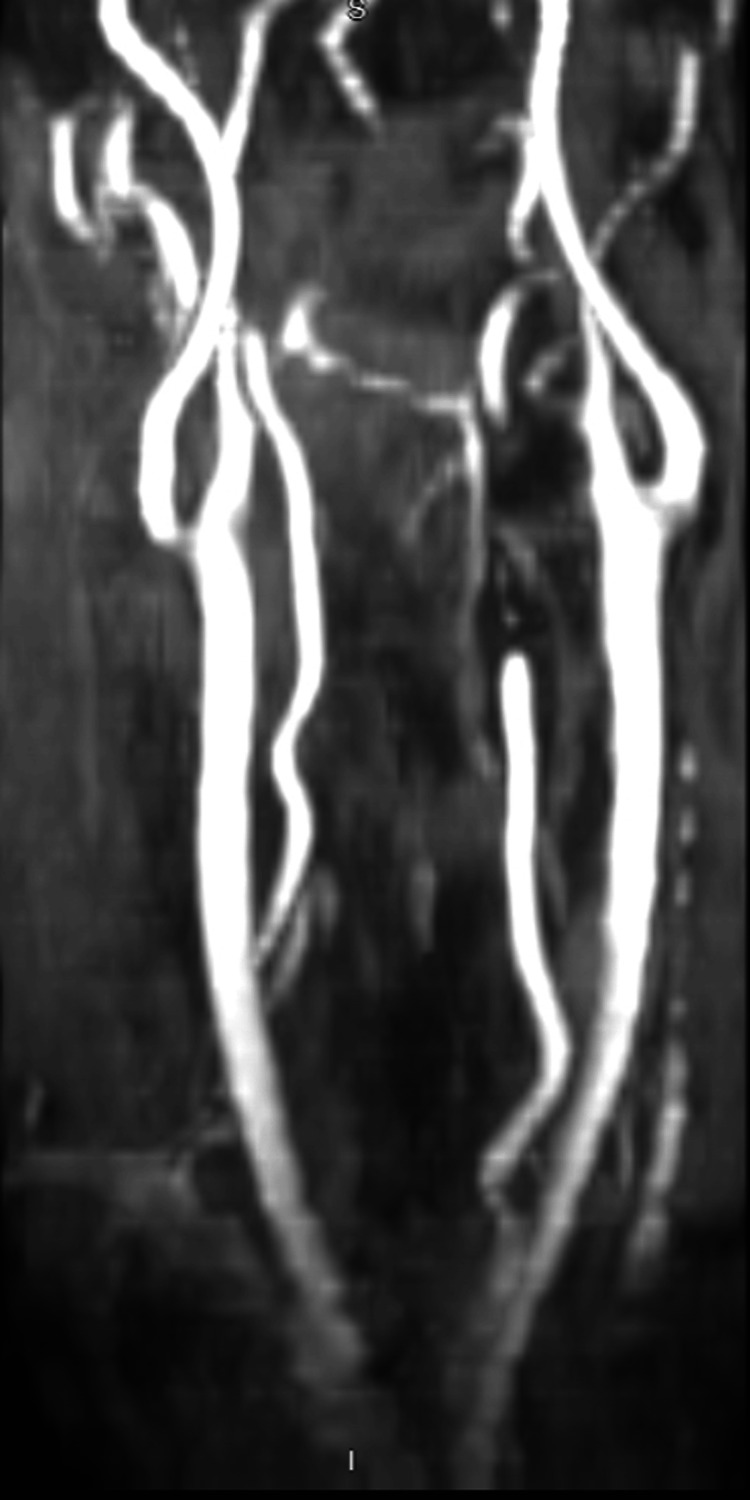
The immediate post-op MRA Immediate post-op MRA following pipeline embolization of the left vertebral artery pseudoaneurysm shows filling of the left V2 (proximal, mid, distal), V3, and V4 segments, along with visualization of the left posterior-inferior cerebellar artery. A giant pseudoaneurysm measuring approximately 4.5 cm x 2.7 cm is noted in the left V2 segment at the C3-C4 level, with increased stasis within the aneurysm. The distal and proximal portions of the device show good wall apposition, while the midportion exhibits approximately 60% luminal narrowing, adjacent to the inflow region of the aneurysm. MRA: magnetic resonance angiography

A 3.5 mm x 25 mm pipeline embolization device was deployed across the pseudoaneurysm, revealing mid-portion narrowing on post-deployment angiograms. Balloon angioplasty with a 6 mm x 15 mm Eclipse balloon improved the narrowing, and a second pipeline embolization device (3.75 mm x 25 mm) was deployed overlapping the first. Follow-up magnetic resonance angiography showed significant pseudoaneurysm stasis and good device apposition, with no evidence of intracranial hemorrhage or thromboembolic complications (Figure 3). Catheters were removed, and hemostasis was achieved with an Angioseal device. The patient was advised to continue taking aspirin 81 mg and prasugrel 10 mg daily, and a cervical spine MRI was ordered. Additionally, the patient was admitted to the neurointensive care unit for close monitoring.

**Figure 4:**
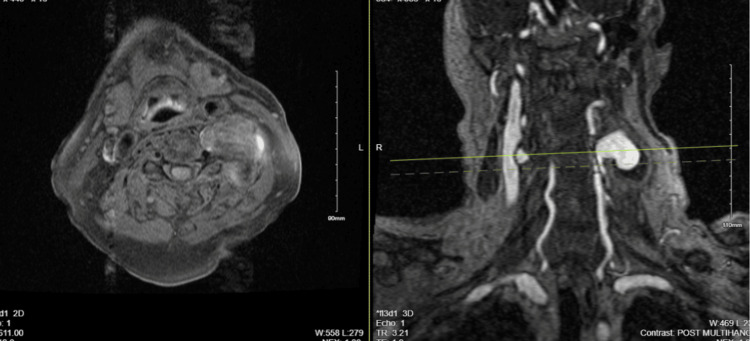
MRA of the neck with and without contrast, post-stent embolization The MRA shows normal flow enhancement distally in the left vertebral artery. Residual blood products are seen emanating from the left vertebral foramen and epidural space at C3-C4 into the excluded pseudoaneurysm sac within the left paravertebral soft tissues. No intrinsic T1 hyperintense signal is present along the course of the left vertebral artery, ruling out dissection.

The MRI showed a mildly expansile T2/Short Tau Inversion Recovery (STIR) hyperintense signal in the right ventral spinal cord at C3-C4, suggesting a focal spinal cord infarct or edema, likely linked to vascular intervention. The left V2 vertebral artery pseudoaneurysm was confirmed, along with multilevel cervical spondylosis and significant foraminal stenosis at C3-C4 and C5-C6, and moderate stenosis at C6-C7. No high-grade spinal canal stenosis was found (Figure 1). A follow-up magnetic resonance angiogram revealed normal carotid, subclavian, and vertebral arteries with no significant stenosis. Post-stent embolization of the left V2 pseudoaneurysm showed residual blood products extending into the left paravertebral and epidural spaces at C3-C4. Delayed filling of the aneurysm sac suggested a possible residual connection, though stent artifacts may limit the evaluation (Figure 4).

The patient remained stable post-procedure and was transferred to an acute rehab facility on Day 8 with controlled pain and no complications. At the final follow-up, he showed improved left arm strength and mobility, with imaging revealing residual blood products but no new dissection. He was advised to continue dual antiplatelet therapy for six months, then switch to aspirin monotherapy. At the one-year follow up, the patient was doing well and had returned to all normal activities without limitations, though he did retain some residual 4/5 left deltoid strength.

## Discussion

This case study presents a rare and complex presentation of a traumatic vertebral artery pseudoaneurysm in a 69-year-old male, underscoring the diagnostic challenges and the unique treatment plan. Vertebral artery pseudoaneurysms from cervical trauma are subtle and uncommon, often mimicking more common conditions such as cervical radiculopathy or musculoskeletal injuries, leading to treatment delays [2]. In this case, the pseudoaneurysm likely formed after the patient’s fall on the right side, which caused hyperextension of the left neck, resulting in trauma to the lateral wall of the left vertebral artery. The presentation, combined with imaging findings that initially suggested a musculoskeletal condition, reflects the complexity of diagnosing pseudoaneurysms, which often present with nonspecific symptoms.

Given the patient's bilateral upper extremity symptoms, it is likely that the subjective weakness and pain on the right side were due to the initial injury and subsequent right-sided cord edema at the C6 level. In contrast, the left-sided weakness in the biceps and deltoid muscles was likely due to the mass effect of the large pseudoaneurysm impinging on the roots of the musculocutaneous and axillary nerves. This case demonstrates how even minor trauma can lead to significant neurological deficits, highlighting the complex interplay between vascular and neurological structures in managing cervical pseudoaneurysms, which, though typically linked to high-energy trauma, can pose diagnostic and treatment challenges in low-energy injuries.

Traditionally, treatment options for vertebral pseudoaneurysms have included surgical resection or endovascular techniques, such as coil embolization [10-12]. The decision to use pipeline embolization reflects a shift towards more advanced endovascular approaches that offer advantages in terms of reduced procedural risk and improved long-term outcomes [13-16]. The successful application of this technique in our patient, with subsequent imaging showing significant stasis and no major complications, supports the potential for pipeline embolization to become a viable option in similar cases.

The patient's postoperative recovery and ongoing rehabilitation also highlight important considerations. The integration of a multidisciplinary approach involving vascular surgery, neurosurgery, and neurology was crucial in addressing both the vascular injury and its neurological implications. The emphasis on rehabilitation and gradual resumption of activities reflects an understanding of the complexities involved in managing such cases, as noted in other reports emphasizing the need for comprehensive follow-up and tailored rehabilitation strategies [17-18].

Other reported cases of pseudoaneurysms highlight important differences in mechanism, location, and treatment approach. For instance, the cases by Dolati et al. (2015) and Ahmadinejad et al. (2023) both involve high-energy trauma leading to pseudoaneurysms: one from a penetrating injury to the carotid artery and the other from deceleration trauma causing a thoracic aortic pseudoaneurysm [5,19]. Similarly, another case focused on endovascular treatment of a carotid pseudoaneurysm with covered stents, demonstrating a distinct treatment strategy due to the aneurysm’s location in a high-flow artery [20]. While these cases share the complexity of managing pseudoaneurysms, they involve higher-energy trauma, different anatomical regions, and alternative treatment approaches, distinguishing them from the cervical pseudoaneurysm seen in our patient, which was successfully managed with pipeline embolization from a low-energy trauma.

## Conclusions

This case underscores the diagnostic and management complexities of a vertebral artery pseudoaneurysm, a rare condition resulting from relatively low-energy trauma. The application of pipeline embolization in this case illustrates a progressive treatment approach, enhancing both procedural safety and long-term results. The case emphasizes the critical role of a multidisciplinary team and diverse rehabilitation in achieving optimal recovery. Unlike other cases involving higher-energy injuries and different vascular locations, this scenario highlights the distinct challenges associated with cervical pseudoaneurysms and the specific considerations required for their effective management.
